# Prolonged versus brief balloon inflation during arterial angioplasty for de novo atherosclerotic disease: a systematic review and meta-analysis

**DOI:** 10.1186/s42155-019-0072-2

**Published:** 2019-08-17

**Authors:** Mark Rockley, Prasad Jetty, Aleksandar Radonjic, Kathleen Rockley, George Wells, Dean Fergusson

**Affiliations:** 1 Division of Vascular and Endovascular Surgery, Department of Surgery, University of Ottawa, The Ottawa Hospital - Civic Campus, Ottawa, K1Y4E9 Canada; 2 Cardiovascular Research Methods Centre, University of Ottawa Heart Institute, Ottawa, K1Y4W7 Canada; 3 Clinical Epidemiology Program, Ottawa Hospital Research Institute, Ottawa, K1H8L6 Canada

**Keywords:** Endovascular, Angioplasty, Duration, Prolonged inflation, Stenosis, Residual stenosis

## Abstract

**Objective:**

Angioplasty is a fundamental treatment for atherosclerotic disease and may be performed as the sole therapy in small vessel disease. However, the ideal duration of balloon inflation has not yet been identified. Our study investigated whether prolonged inflation of at least 1-min duration, when compared with brief inflation, affects residual stenosis after arterial angioplasty.

**Data sources and methods:**

Two independent reviewers conducted a systematic review of EMBASE, MEDLINE, CENTRAL, trial registries and grey literature, using pre-specified search syntax. Data abstraction and quantitative analysis was performed independently, according to pre-specified criteria. The primary outcome was residual stenosis after initial angioplasty, in addition to other pre-specific clinical and radiographic outcomes. All analyses were stratified by coronary, cerebrovascular, and peripheral territory. The study protocol is published and registered on PROSPERO (CRD42018092702).

**Results:**

Six relevant articles were identified, of which one investigated peripheral vascular angioplasty and five investigated coronary artery angioplasty, encompassing 1496 procedures. The studies were at moderate risk of bias. Minimal heterogeneity within coronary studies allowed for subgroup meta-analysis. Prolonged inflation was significantly associated with lower risk of residual stenosis post-inflation in the pooled coronary trials (RR 1.76 [95% CI 1.46–2.12], I^2^ = 0%, *p* < 0.001) in addition to approaching significance in the peripheral vascular trial (RR 2.40 [95% CI 0.94–6.13], *p* = 0.07). Prolonged inflation was associated with less risk of arterial dissection and need for adjunctive procedures such as stenting. Following adjunctive procedures, less residual stenosis was still observed in the prolonged angioplasty group in the reported coronary studies. Follow-up data did not reveal a significant difference in the presence of restenosis, however there was a long-term benefit of prolonged inflation in reducing overall severity of stenosis.

**Discussion:**

This is the first review investigating outcomes related to duration of balloon inflation. Both coronary and peripheral vascular evidence are in agreement that prolonged angioplasty balloon inflation greater than 60 s appears to be associated with improved immediate post-inflation results. However, long-term data is heterogeneous and inconsistently reported. We propose further investigation to address outstanding long-term outcomes, particularly in small vessel territories such as tibial vessels where angioplasty is often used as the only endovascular therapy.

**Trial registration:**

This protocol has been registered with the International Prospective Register of Systematic Reviews (PROSPERO: CRD42018092702) prior to conduct of the review.

**Electronic supplementary material:**

The online version of this article (10.1186/s42155-019-0072-2) contains supplementary material, which is available to authorized users.

## Background

Atherosclerotic vascular disease is a chronic disease that can affect multiple organs, and is collectively the most common cause of death in North America. Angioplasty is a foundational treatment of endovascular therapy in coronary, cerebral, and peripheral vascular territories. While angioplasty may be augmented by treatments such as stenting or atherectomy in larger vessels, the feasibility of adjunctive procedures is limited in small vessel disease. Unfortunately, 20–50% of atherosclerotic peripheral vascular lesions treated with plain balloon angioplasty still experience primary failure within two years, depending on lesion location and characteristics. (Lazaris et al. 2004).

Various techniques have been proposed to minimize restenosis following angioplasty, however many have not been thoroughly evaluated in randomized trials. One such technique is prolonged angioplasty balloon inflation time, which is theorized to reduce post-interventional dissection and induce smooth muscle dysfunction, thereby reducing vasospasm and resulting stenosis. While animal studies have not revealed short-term mechanical advantage for prolonged balloon inflation (Labinaz et al. 1999), smooth muscle dysfunction has been observed (Consigny & LeVeen 1988). Alternatively, prolonged balloon inflation may theoretically incur risk to the patient by simply masking flow-limiting dissections, which may have only been identified if transient balloon inflation was used. This theory is supported by a recent study which detected over twice as many dissections in the brief inflation group when compared with the prolonged inflation group, while half of the dissections initially observed in the brief inflation group were no longer appreciable after repeated balloon inflation (Horie et al. 2018). Identification of these dissections is relevant as they are at high risk of causing target lesion occlusion, and may necessitate stent placement.

Conversely, in addition to being a therapeutic treatment for atherosclerotic disease, balloon angioplasty is known to cause vascular damage to previously healthy arteries, and is used in animal studies to induce traumatic stenosis. This contradiction raises concern that although angioplasty may result in aesthetic immediate results, there is a fundamental limitation to preserving long-term results after angioplasty. In particular, prolonged angioplasty balloon inflation may result in superior immediate results, but may also cause more profound vascular injury. The potential risks to patients of long-term vascular injury due to prolonged angioplasty are currently unknown.

Early randomized trials suggested immediate benefit of prolonged balloon inflation, although the sustainability of the patency benefit is conflicting (Arie et al. 1990; Garrahy et al. 1991; Hovasse et al. 2013; Umeda et al. 2004). However, the immediate benefit of prolonged angioplasty has not been consistently reported in randomized trials (Zorger et al. 2002). Despite the ubiquitous use of angioplasty in atherosclerotic disease and ongoing primary investigation, there are no systematic reviews evaluating prolonged angioplasty balloon inflation. Currently synthesized evidence is insufficient to confidently direct clinical decision-making, and the current variation in operator preference of balloon angioplasty duration suggests ongoing clinical equipoise.

The purpose of this systematic review was to evaluate the risks and benefits of prolonged angioplasty balloon inflation duration in the diverse patients with atherosclerotic disease. Specifically, our primary objective was to determine whether prolonged balloon inflation of at least 1-min duration, when compared with more brief balloon inflation, affects incidence of residual stenosis in the immediate post-inflation angiogram after arterial angioplasty for atherosclerotic disease.

## Methods

This study is registered on PROSPERO (CRD42018092702) and an a priori protocol has been published (Rockley et al. 2019). A systematic review was performed using independent reviewers. EMBASE, MEDLINE, and CENTRAL databases were searched with a pre-specified search strategy generated with assistance of a health sciences librarian (Additional file 1: Appendix 1). ClinicalTrials.gov registry was searched for trial registry data, and OpenGrey was interrogated for unpublished relevant literature. Following an initial abstract screen, full-text review of potentially relevant articles was conducted. To ensure capture of all relevant trials, all eligible studies also underwent ancestry search, in addition to citation search using SCOPUS. All discovered studies were aggregated into EndNote X8.2, before undergoing screening using DistillerSR. Data abstraction was performed independently by both reviewers, and any disagreements were addressed by a third reviewer. Finally, RevMan 5.3 was used for data analysis and synthesis. All studies were assessed for risk of bias using the Cochrane Collaboration tool.

Eligible study designs included randomized controlled trial (RCT), cluster RCT, non-randomized controlled trials, cluster trials, interrupted time series studies, and controlled before-after studies (CBA) which compared prolonged balloon inflation of at least 1-min duration with more brief balloon inflation. The threshold of 1-min inflation duration is based on rabbit model studies which demonstrated balloon inflation of at least 1 min is associated with inhibitory effect of mesenchymal stem cells and medial necrosis leading to intimal hyperplasia (Doornekamp et al. 1996; Kim et al. 2016). The search included studies examining human adults (age 18 or older) who received angioplasty for atherosclerotic stenotic or obstructive vascular disease. English and French studies published between January 1977 – February 2018 were considered. The start year of 1977 was chosen as the first in-human use of angioplasty was performed that year (Gruentzig 1977).

Studies in which angioplasty was the primary purpose of the intervention were included, while those performed concurrently with a hybrid open vascular procedure on an in-line flow artery, such as an endarterectomy or bypass, were excluded. We included interventions performed on any arterial structure – including coronary, extracranial, intracerebral, or extremity arteries – with balloons either drug-coated or lined with cutting ribs. Studies were stratified by vascular bed for analysis. Studies were excluded if they employed adjunctive endovascular procedures prior to measurement of the outcome of interest, including but not limited to: stent placement, orbital atherectomy, laser atherectomy, rotational atherectomy, or directional atherectomy. Venous angioplasty, arteriovenous fistula angioplasty, and studies examining emergency settings were also excluded. Studies only examining de-novo stenosis were included, as the histopathology and outcomes related to restenosis are vastly different than de-novo atherosclerosis (Mittal et al. 1997).

The primary outcome of interest was the risk ratio of residual stenosis immediately following angioplasty, as determined by post-inflation angiogram. The definition threshold for residual stenosis must have been explicitly defined for study inclusion. In addition to the incidence of restenosis immediately after balloon inflation, secondary outcomes included the percentage of restenosis, reported as a difference in means. We noted if adjunctive procedures such as further angioplasty or stenting were required, as well as residual stenosis following these adjunctive procedures. Accessory radiographic adverse events were recorded; this excluded residual stenosis, and was defined as a vascular anatomic abnormality that did not exist prior to balloon inflation. This included arterial dissection, vessel perforation, and acute occlusion where there was a degree of patency prior to intervention. In addition to radiographic adverse events, newly developed clinically significant adverse events occurring during the day of intervention were noted. This broadly encompassed ischemic symptoms secondary to the territory of vascular intervention: for instance, chest pain or a myocardial infarction following coronary artery angioplasty, stroke following cerebral vessel angioplasty, or acute limb ischemia following peripheral arterial angioplasty.

Long-term secondary outcomes were also differentiated into radiographic or clinical outcomes. These included both incidence and severity of radiographic vessel restenosis, when performed at least 3 months following the initial intervention, using any validated method; interventional angiogram, CT-angiogram, MRI-angiogram, angioscopy, and Duplex ultrasound were considered. Clinically, symptoms indicating long term resolution of ischemia included angina pectoralis, cerebral hypoperfusion syndromes, claudication, or critical limb ischemia.

Following data collection, a meta-analysis with random effects model was performed under pre-specified conditions, stratified by vascular location into peripheral, coronary, and cerebrovascular. Measurement effects were determined by using risk ratio with 95% confidence intervals. The unit of analysis was arterial lesions; one subject could lend multiple units of analyses if they had multiple lesions undergoing angioplasty. Clustering of data for purposes of meta-regression was not considered due to variable reporting patterns, resulting in statistical limitations. Due to the anticipated heterogeneous nature of clinical symptomatic status presentation and reporting, in particular given the broad anatomic inclusion of this study, long-term clinical symptom outcomes were not assessed using quantitative meta-analysis.

## Results

**Fig. 1:**
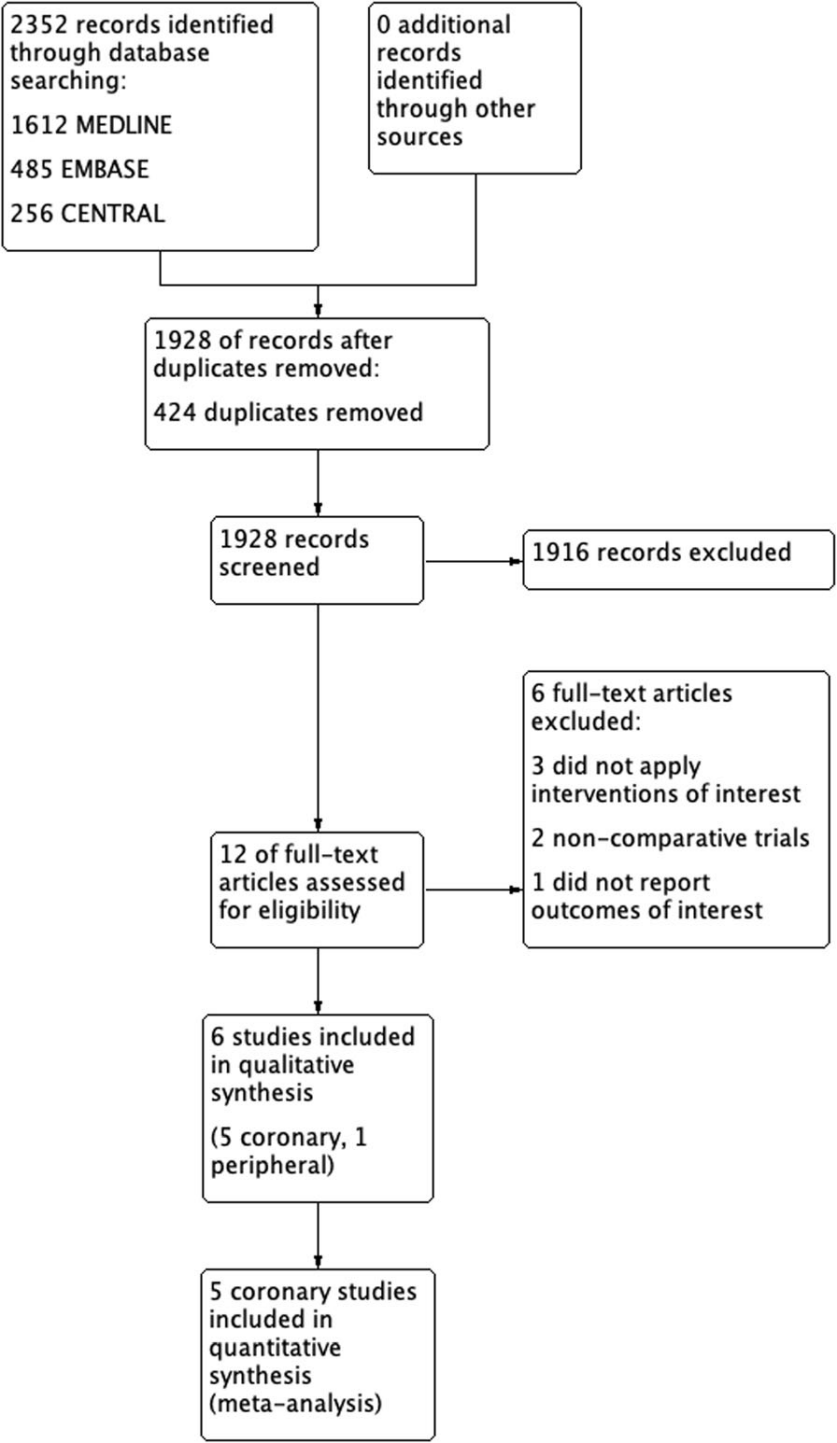
Systematic review PRISMA study flow diagram. In total, the 6 studies included in the qualitative and quantitative analysis included 1496 procedures

Following systematic searching of the named databases by two independent reviewers, 2352 articles were identified, totalling 1928 after removal of duplicates (Fig. 1). Title and abstract screening identified 12 articles that were potentially relevant to our review. Full text screening confirmed that six articles met our eligibility criteria, while the other six articles were excluded due to reasons detailed in Additional file 1:Appendix 2. Of the six articles that were included in analysis, five were randomized control trials (Cribier et al. 1995; Eltchaninoff et al. 1996; Ohman et al. 1994; Umeda et al. 2004; Zorger et al. 2002), and one was an institutional before-after trial (Arie et al. 1990). One study investigated femoropoliteal arterial interventions (Zorger et al. 2002), while the other five investigated coronary interventions. No study investigated cerebrovascular territories. The six studies included a total of 1496 procedures. While there was variation in study protocol between the prolonged inflation definition (60 to 900 s), there was more consistency between studies in the definition of brief inflation duration (30 to 60 s). Only the before-after trial (Arie et al. 1990) allowed for less than a 2:1 ratio in total initial inflation duration between prolonged and brief inflation. Four trials allowed for multiple inflations (Arie et al. 1990; Cribier et al. 1995; Eltchaninoff et al. 1996; Ohman et al. 1994), while the other two trials (Umeda et al. 2004; Zorger et al. 2002) only protocoled a single inflation. Study design and characteristics are summarized in Additional file 1: Appendix 3.

The immediate post-inflation radiologic and clinical outcomes are presented in Additional file 1: Appendix 4. The mean reported total balloon inflation times after all treatments ranged from 101 to 198 s and 238.8–973 s in the brief and prolonged inflation groups, respectively. Based on individual definitions of residual stenosis from each trial, which ranged from 30 to 50%, all studies noted a lower incidence and/or severity of residual stenosis in the prolonged inflation groups. Furthermore, all reporting trials noted less incidence of dissections in the prolonged inflation groups. Reporting of immediate clinical outcomes was inconsistent; however, three coronary trials noted an increased incidence of inability to tolerate the entire duration of balloon inflation in the prolonged inflation groups (Eltchaninoff et al. 1996; Ohman et al. 1994; Umeda et al. 2004).

Five trials (Arie et al. 1990; Eltchaninoff et al. 1996; Ohman et al. 1994; Umeda et al. 2004; Zorger et al. 2002) reported off-protocol adjunctive procedures performed after initial angioplasty, which consisted of either further balloon inflation or use of stent (Additional file 1: Appendix 5). The use of adjunctive procedures was less common in the prolonged inflation groups for each trial, and following adjunctive procedures, less residual stenosis was still observed in the prolonged angioplasty groups. It should be noted that no study blinded the operator to the group allocation, therefore decisions to perform adjunctive procedures were subject to potential bias.

Three studies (Eltchaninoff et al. 1996; Ohman et al. 1994; Umeda et al. 2004) also reported long-term outcomes, all in coronary arteries, and ranging from 4 to 12 months after the index procedure (Additional file 1: Appendix 6). All long-term anatomic surveillance was performed with angiogram and was performed on the majority (66% - 96%) of study participants in each trial. Although the incidence of stenosis during long-term follow-up was inconsistent among trials, all three reported a decrease in mean continuous severity of stenosis. Of note, no study defined how the index target lesion was identified, rather than further target vessel stenosis in a location other than the initial target lesion. Reporting of long-term clinical outcomes was inconsistent and did not specifically reference the preoperative status of individual patients.

Because the coronary vascular bed was the only vascular territory with multiple reported trials, statistical pooled analysis was performed within the coronary subgroup. Acceptable heterogeneity among reports of the primary outcome and several secondary outcomes in coronary arteries allowed for meta-analysis. The single peripheral vascular study is presented separate but alongside the pooled estimates of the coronary subgroup.

**Fig. 2:**
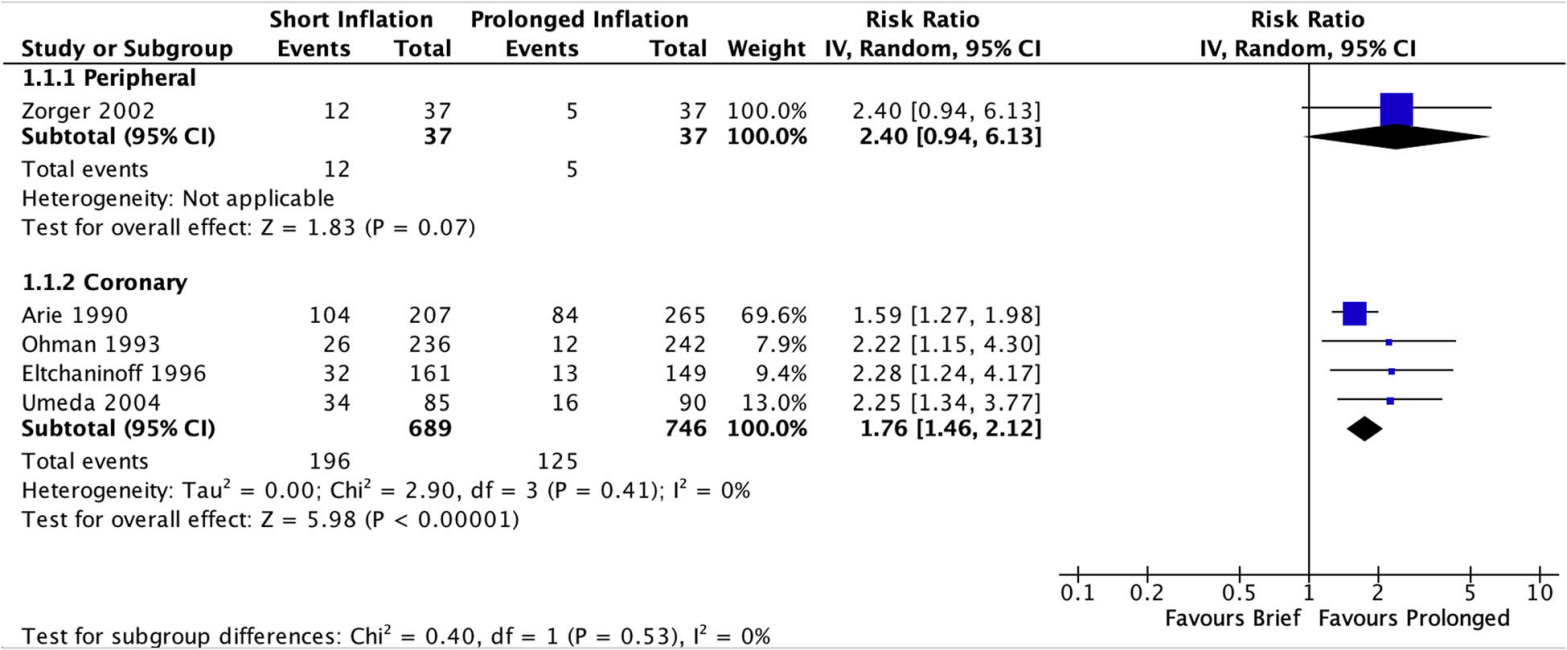
Risk of residual stenosis immediately after initial angioplasty inflation

**Fig. 3:**
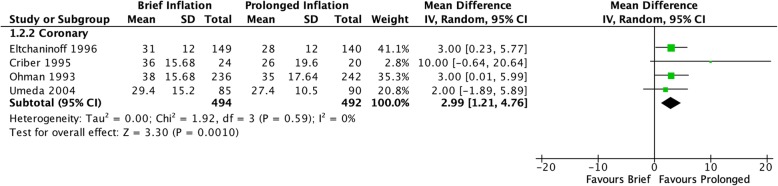
Severity of residual stenosis after angioplasty inflation, measured as a continuous outcome of percent stenosis

In the coronary studies, brief balloon inflation was significantly associated with greater risk of residual stenosis immediately post-inflation (RR 1.76 [95% CI 1.46–2.12], I^2^ = 0%, Fig. 2) and greater severity of residual stenosis (MD 2.99 [95% CI 1.21–4.76], I^2^ = 0%, Fig. 3). The single peripheral vascular study found a trend towards increased incidence of residual stenosis after brief inflation immediately post-inflation that did not reach significance (RR 2.40 [95% CI 0.94–6.13], *p* = 0.07).

**Fig. 4:**
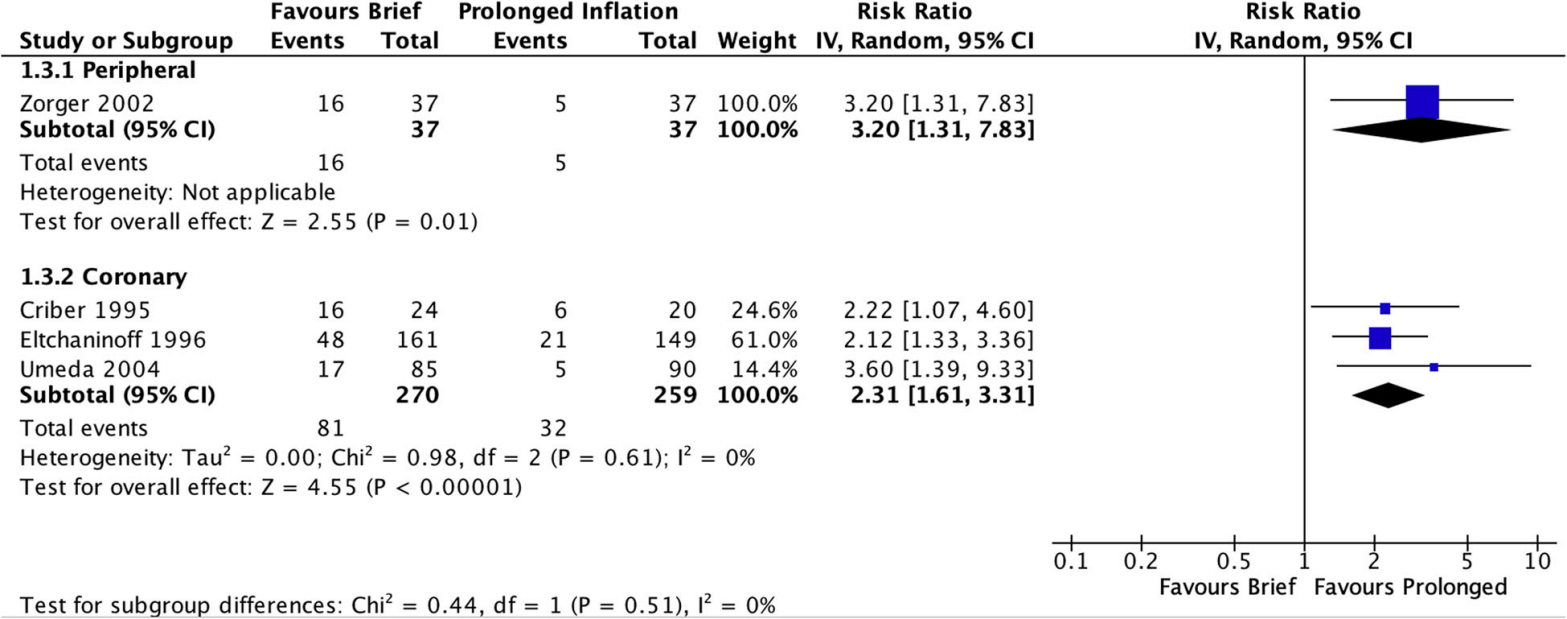
Risk of arterial dissection apparent immediately after initial angioplasty inflation

In addition to residual stenosis, brief balloon inflation was also associated with greater risk of arterial dissection in the pooled coronary studies (RR 2.31 [95% CI 1.61–3.31], I^2^ = 0%, Fig. 4) as well as the single peripheral vascular study (RR 3.20 [95% CI 1.31–7.83]).

**Fig. 5:**
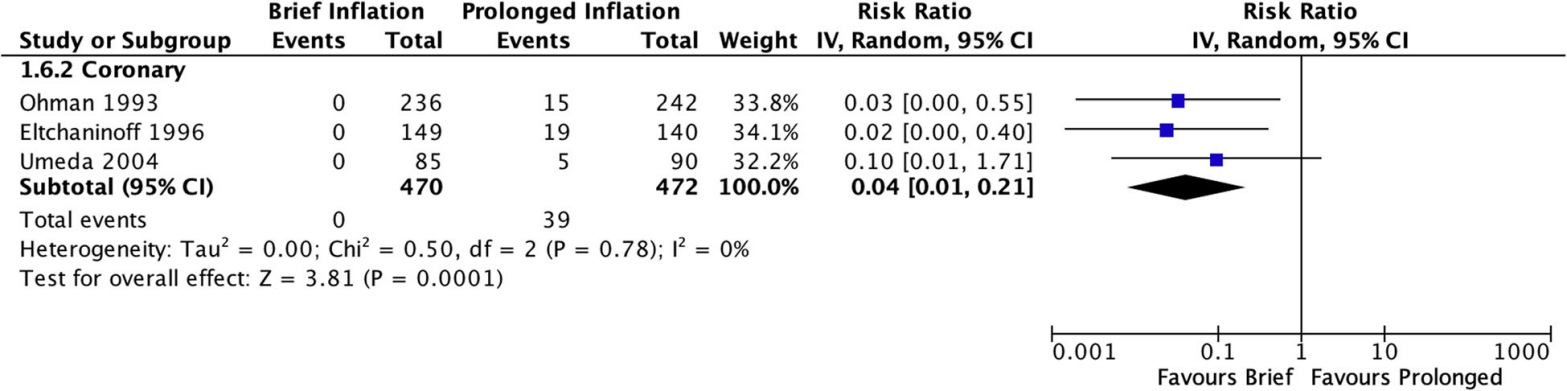
Risk of patient inability to tolerate initial duration of balloon inflation due to end-organ ischemia

Clinically apparent end-organ ischemia during inflation was only reported in coronary studies, in the form of chest pain or ischemic ECG changes, and was significantly less common during brief angioplasty (RR 0.04 [95% CI 0.01–0.21], I^2^ = 0%, Fig. 5).

**Fig. 6:**
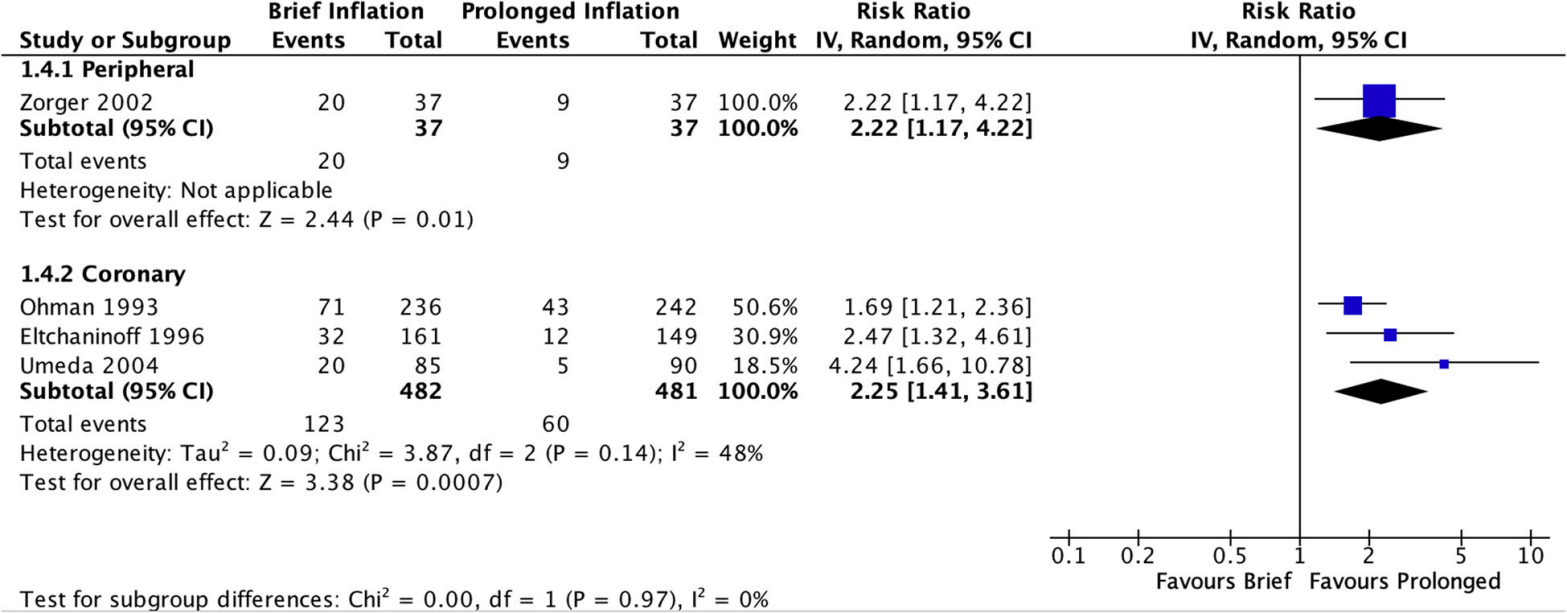
Risk of receiving adjunctive procedures performed after initial inflation, during the same operative setting

**Fig. 7:**
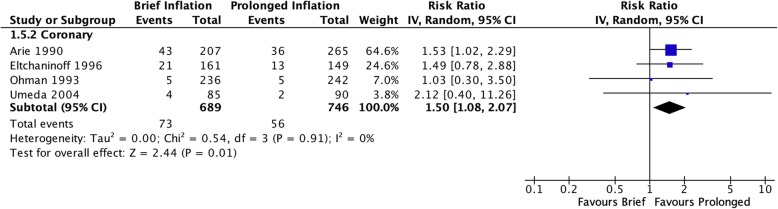
Risk of residual stenosis on index procedure completion angiogram, following any adjunctive procedures

**Fig. 8:**
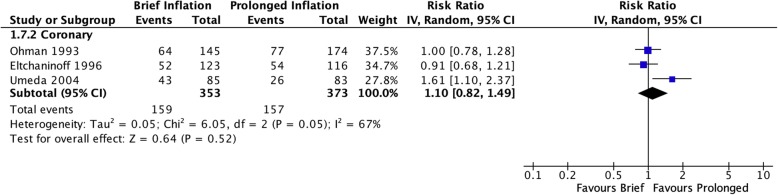
Risk of stenosis on follow-up angiogram performed at least 3 months following index procedure

**Fig. 9:**
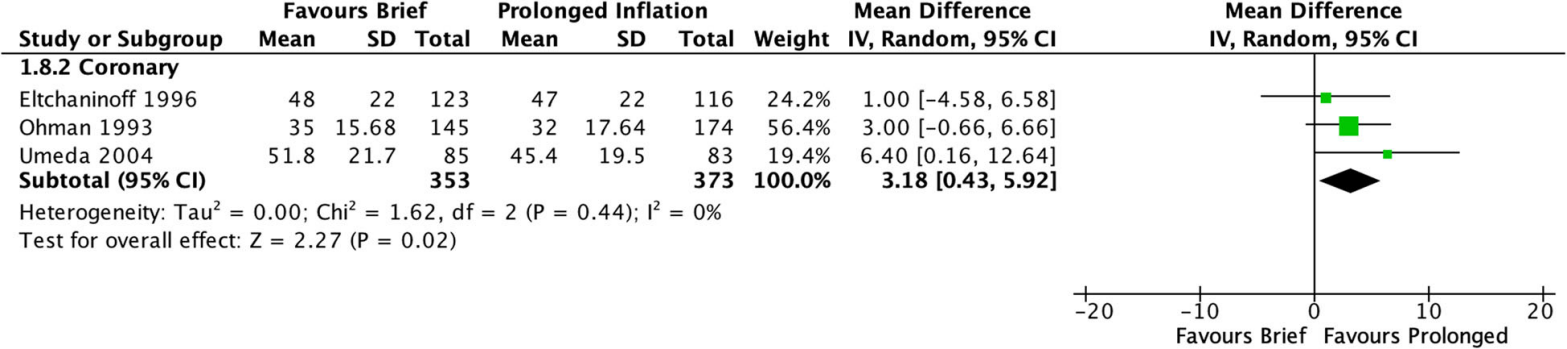
Severity of stenosis on follow-up angiogram performed at least 3 months following index procedure, measured as a continuous outcome of percent stenosis

Off-protocol adjunctive procedures such as repeat angioplasty and/or stenting were more likely to be performed after brief inflation in the pooled coronary studies (RR 2.25 [95% CI 1.41–3.61], I^2^ = 24%, Fig. 6) as well as in the single peripheral study (RR 2.22 [95% CI 1.17–4.22]). Only coronary studies reported residual stenosis following adjunctive procedures at the completion of the procedure, which demonstrated a persistent increased risk of residual stenosis after initial brief inflation (RR 1.50 [95% CI 1.08–2.07], I^2^ = 0%, Fig. 7). Only three coronary studies (Eltchaninoff et al. 1996; Ohman et al. 1994; Umeda et al. 2004) reported long-term restenosis risks. Pooled analysis of these studies did not reveal a significantly increased risk of brief inflation on the binary presence of restenosis (RR 1.10 [95% CI 0.82–1.49], I^2^ = 67%, Fig. 8), however there was a benefit associated with prolonged inflation in reducing overall severity of stenosis (MD 3.18 [95% CI 0.43–5.92], I^2^ = 0%, Fig. 9).

**Fig. 10:**
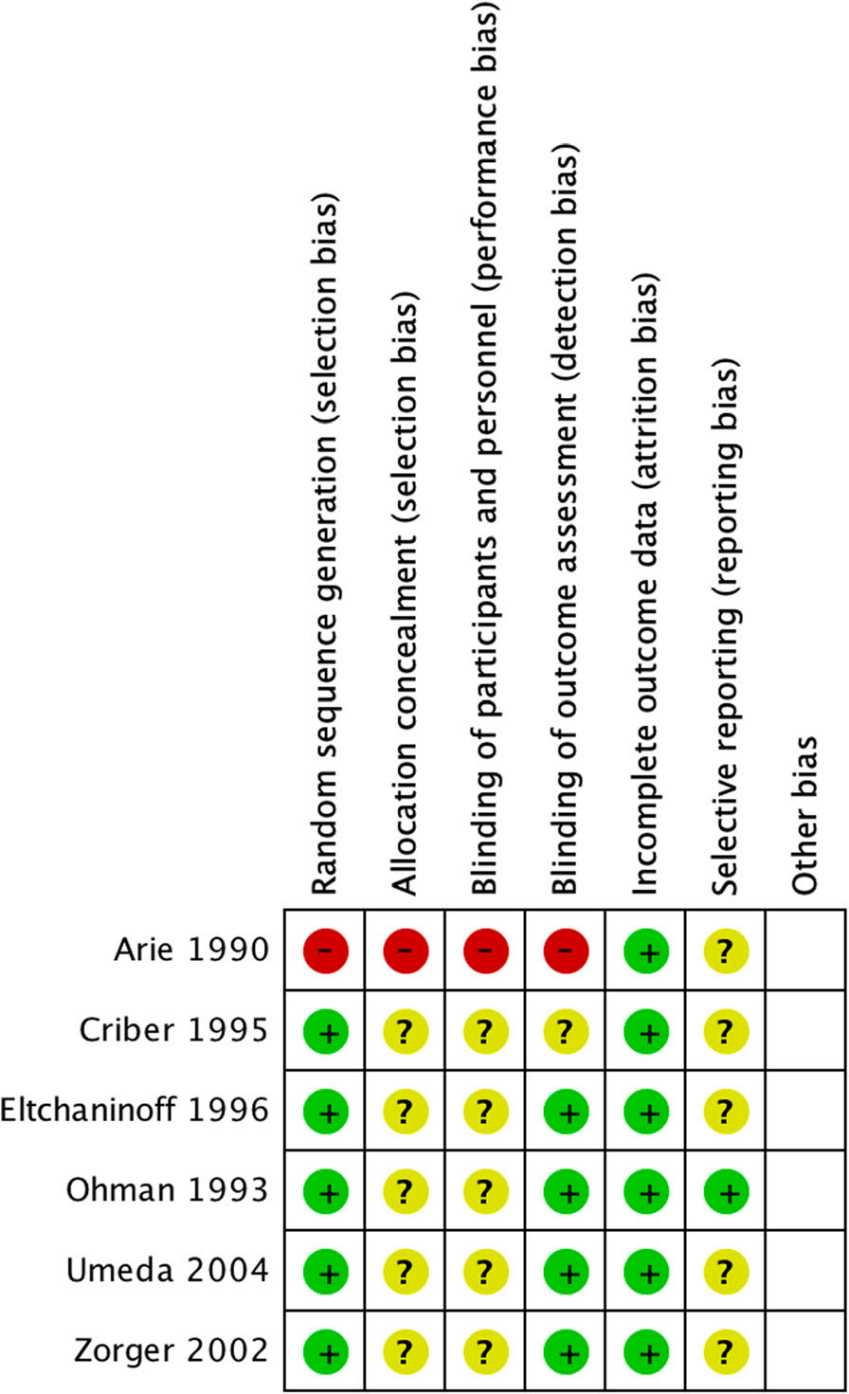
Individual Study Risk of Bias. In accordance with Cochrane Guidelines, each included study was individually assessed for risk of bias

**Fig. 11:**
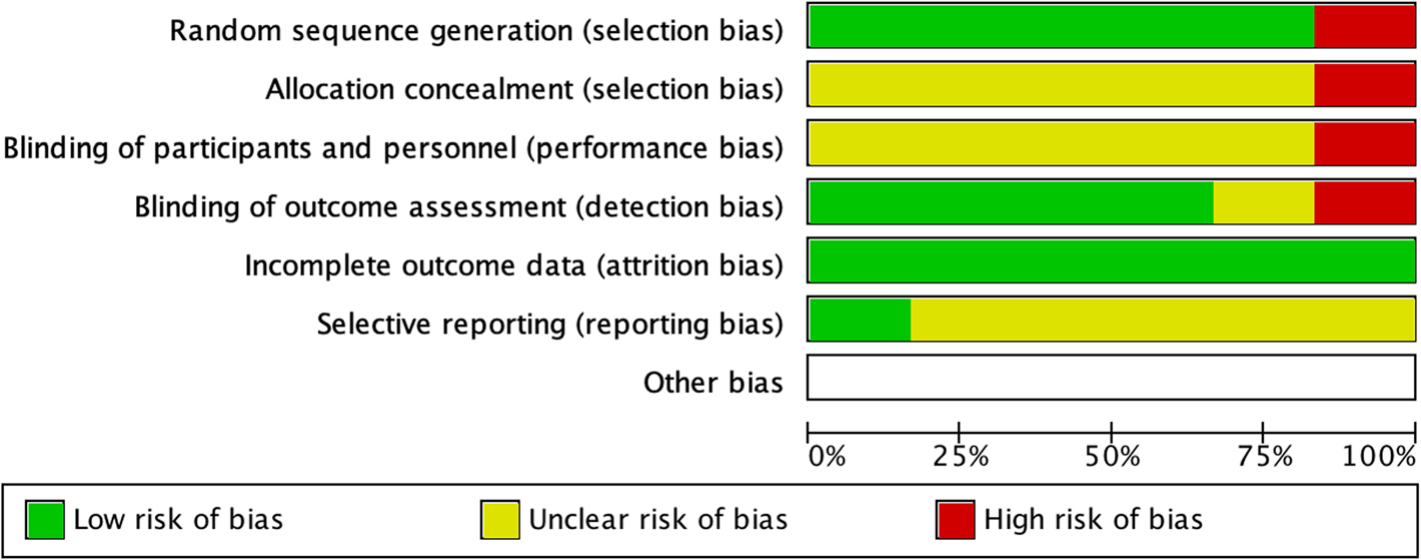
Summary of Risk of Bias. Overall risk of bias in studies included within the review

**Fig. 12:**
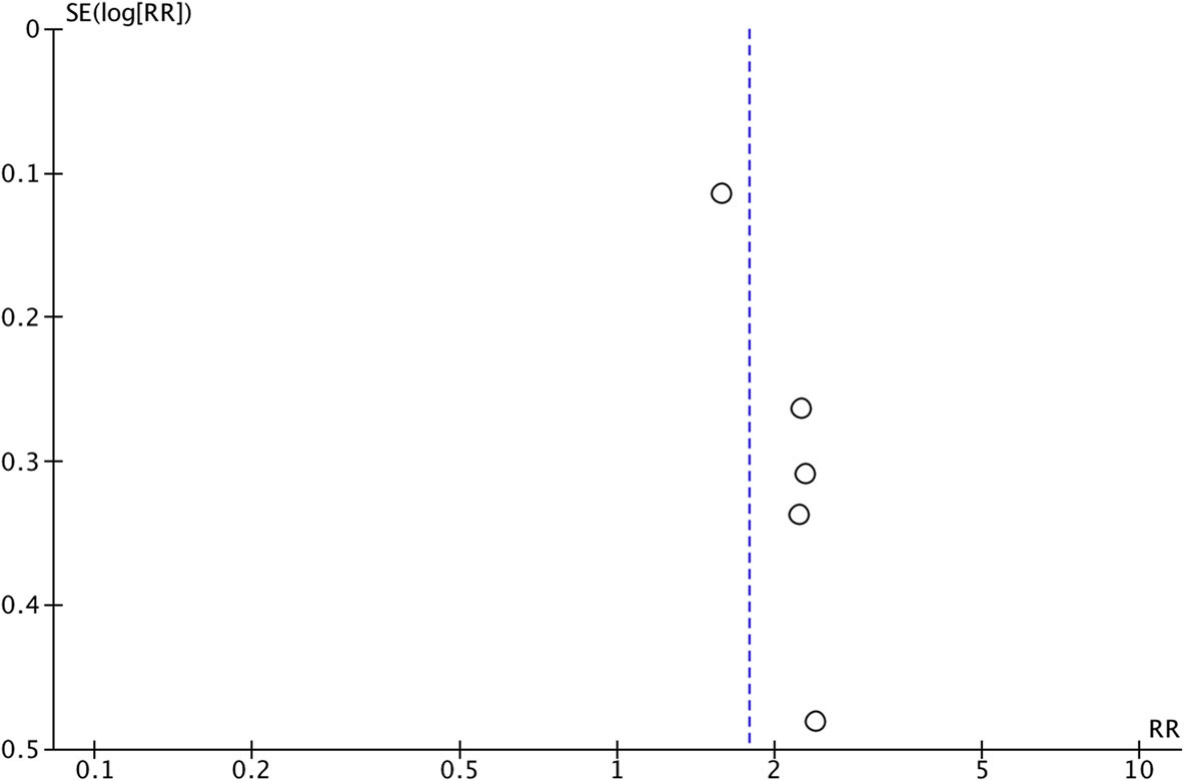
Funnel plot summarizing the five studies reporting on the primary outcome. The vertical dotted line indicates the overall pooled effect measure (Relative Risk)

Each study was assessed for risk of bias, which is summarized by the Cochrane Risk of Bias tool (Figs. 10 and 11). The before-after trial was at greatest risk of bias (Arie et al. 1990), however most trials may have been at risk of selection, reporting, and performance bias. The studies were also considered as individual units of analysis and plotted on a funnel plot, comparing the effect measure (risk ratio) of the primary outcome with an index of precision (Fig. 12). Using the overall pooled effect measure of the primary outcome as a reference, the paucity of low weight publications reporting relatively low effect measure was noted. This is potentially consistent with publication bias, but must also be interpreted with caution as only five studies reporting primary outcomes were included in this analysis. Sensitivity analysis investigating the effect of studies at high risk of bias and non-randomized trials did not reveal significant differences in the pooled effect estimates. Meta-regression could not be performed due to the low number of included trials.

## Discussion

This review identified six studies, including five randomized trials of moderate quality, that investigated the effect of prolonged angioplasty on radiographic and clinical outcomes. Five identified studies investigated coronary arteries, a single study investigated peripheral vascular disease, and no studies investigated cerebrovascular disease. Although protocols varied, all studies explicitly defined and distinguished between brief and prolonged balloon inflation and were associated with good outcome homogeneity for short-term outcomes. Immediate outcomes appeared to favour prolonged balloon inflation, but long-term results were less clear.

Our analysis suggests that immediately post-procedure, brief balloon inflation is associated with a greater risk of residual stenosis, stenosis severity and arterial dissection than prolonged inflation in both peripheral and coronary vascular territories. Evidence suggests that temporary vasospasm may be observed immediately after brief balloon inflation and can mimic residual stenosis (Fischell 1990); however, vasospasm alone would not explain the observed difference in arterial dissection incidence between groups. Thus, other causes may be at play. Possibilities include a slower inflation rate leading to less intimal tearing during inflation, or more adherent wall apposition resulting in a less apparent intimal tear on angiogram (Hollman et al. 1989).

While the brief inflation group protocols all prescribed inflation durations less than 60 s, the actual balloon inflation duration in these groups ranged from 101 to 198 s. The divergence from protocol in the balloon duration of the brief inflation groups suggests that brief balloon inflation may result in insufficient immediate results, resulting in operator decisions to repeat or prolong the inflation.

Adjunctive procedures were performed less frequently after prolonged balloon inflation, which is expected given superior immediate outcomes associated with the technique. However, by potentially not observing fragile lesions that could be prone to observed dissection during brief inflation, prolonged inflation may in fact mask a fragile lesion that will progress to restenosis or occlude post-procedure. While repeat prolonged balloon inflation is often used to treat iatrogenic angioplasty-related arterial dissection with good immediate angiographic results, the long-term durability of this practice is unknown. Recent studies using intravascular ultrasound have demonstrated that subtle arterial dissection is indeed more common than what is observable on angiogram (Spiliopoulos et al. 2019). The long-term impact of minor dissections that are not appreciated on angiogram is unclear. Consequently, these fragile lesions may have benefited from adjunctive procedures such as stent placement, which may then explain the loss of benefit of prolonged inflation in long-term follow-up. The lack of sustained long-term benefit may also be explained by traumatic intimal hyperplasia secondary to prolonged inflation; however, contemporary results of this pathophysiology are largely limited to animal models and thus comparable conclusions to human vasculature remain anecdotal (Consigny & LeVeen 1988).

There are several limitations of this study. None of the included studies specifically distinguished progression of target vessel atherosclerosis from the specific target lesion patency at follow-up. Therefore, progressive disease elsewhere in the artery could be confounding the observed results. Furthermore, the increased use of stenting in the brief inflation group may also be confounding results. Additionally, no trial reported long-term stenosis outcomes of only patients who did not receive adjunctive procedures. Nevertheless, acceptable heterogeneity in primary and secondary outcome reporting among included studies allowed for quantitative analysis with a sufficiently large number of included procedures. While all included studies prescribed the angioplasty inflation pressure in addition to inflation duration, the actual pressure used was not reported in any included study. This is a potential source of confounding because inflation pressure has also been associated with angioplasty outcomes (Scheinert et al. 2016).

Several randomized trials included in this review were published in the 1990’s, prior to widespread adaptation of CONSORT guidelines. The lack of a-priori reporting of outcomes and the inherent difficulties of performance bias in procedural trials may have introduced bias. Although it is difficult to conclusively identify publication bias due to the relatively low number of trials and homogeneity of the primary outcome, there is a trend that is suggestive of publication bias. As a result, our findings must be considered in light of these potential biases.

## Conclusion

In summary, this review identified five trials investigating coronary arteries and one trial investigating peripheral vascular disease. Both coronary and peripheral vascular evidence is in agreement that prolonged angioplasty balloon inflation greater than 60 s appears to be associated with improved immediate post-inflation results. However, long-term data is difficult to interpret due to multiple confounding factors, heterogeneity, and inconsistent reporting. In light of the ongoing use of angioplasty as the initial treatment in many procedures, and often as the sole treatment in small vessel disease, there is a lack of evidence to guide practitioners. In addition, the exact optimal duration of prolonged balloon inflation is unclear and would benefit from further investigation. Specifically, we propose further investigation in tibial vessels, where angioplasty is often used as the only endovascular therapy and prolonged inflation is likely better tolerated than in the coronary circulation.

## Additional file

Additional file 1: **Appendix 1.** Search Syntax: Search syntax for MEDLINE, using OVID interface. Performed on March 1, 2018. **Appendix 2.** Reasons for study exclusions. **Appendix 3.** Baseline characteristics of each study, including design, balloon inflation duration protocol, and the number of units analyzed. **Appendix 4.** Immediate results following initial balloon angioplasty. Blank fields represent unreported data. **Appendix 5.** Off-protocol intraoperative results of any adjunctive procedures following initial angioplasty. Blank fields represent unreported data. **Appendix 6.** Long-term results following balloon angioplasty. Blank fields represent unreported data. (DOCX 39 kb)

## Data Availability

All data generated or analysed during this study are included in this published article and its supplementary information files.

## Abbreviations

CBA: Controlled Before-After Trial
RCT: Randomized Control Trial
