# Supplementary material for: Prolonged versus brief balloon inflation during arterial angioplasty for de novo atherosclerotic disease: a systematic review and meta-analysis
Source: CVIR Endovasc. 2019 Aug 17;2:29. doi: 10.1186/s42155-019-0072-2 (PMC6966366; doi:10.1186/s42155-019-0072-2)
Supplement: Supplementary file 1 — Appendix 1. Search Syntax: Search syntax for MEDLINE, using OVID interface. Performed on March 1, 2018. Appendix 2. Reasons for study exclusions. Appendix 3. Baseline characteristics of each study, including design, balloon inflation duration protocol, and the number of units analyzed. Appendix 4. Immediate results following initial balloon angioplasty. Blank fields represent unreported data. Appendix 5. Off-protocol intraoperative results of any adjunctive procedures following initial angioplasty. Blank fields represent unreported data. Appendix 6. Long-term results following balloon angioplasty. Blank fields represent unreported data. (DOCX 39 kb) [file 42155_2019_72_MOESM1_ESM.docx]

**Appendices**

| **Angioplasty**  1. ANGIOPLASTY, BALLOON/ or ANGIOPLASTY, BALLOON, CORONARY/ |
| --- |
| 2. (balloon* adj2 angioplast*).tw. |
| 3. (transluminal adj3 arteri* adj3 dilation*).tw. |
| 4. (balloon* adj2 inflat*).tw. |
| **Residual Stenosis**  5. Constriction, Pathologic/ |
| 6. Vascular Patency/ |
| 7. (residual adj2 stenos*).tw. |
| 8. (angiogra* adj2 result*).tw. |
| 9. (unstable adj2 lesion*).tw. |
| 10. (morpholog* adj2 result*).tw. |
| 11. (radio* adj2 result*).tw. |
| **Balloon Inflation Time**  12. Time Factors/ |
| 13. prolonged.tw. |
| 14. duration.tw. |
| 15. brief.tw. |
| 16. (inflation adj2 time*).tw. |
| 17. patency.tw. |
| **Combination of Search Concepts**  18. 1 or 2 or 3 or 4 |
| 19. 5 or 6 or 7 or 8 or 9 or 10 or 11 or 17 |
| 20. 12 or 13 or 14 or 15 or 16 |
| 21. 18 and 19 and 20 |
| **Search Limits**  22. limit 21 to "all adult (19 plus years)" |
| 23. limit 22 to yr="1977 - 2018" |

**Appendix 1: Search Syntax:** Search syntax for MEDLINE, using OVID interface. Performed on March 1, 2018.

| **Study** | **Category of Exclusion** | **Reason for Exclusion** |
| --- | --- | --- |
| **Palazzo**^18^  **1988** | Study Design | Single-arm observational trial; no head-to-head comparison. |
| **Garrahy**^8^  **1991** | Intervention | Inflation duration for prolonged inflation treatment arm was not greater than 60 seconds. |
| **Blankenship**^19^  **1999** | Intervention | Total inflation duration was not compared in this trial, but instead rapidity and frequency of balloon inflation. |
| **Söder**^20^  **2002** | Study Design | Single-arm trial; no head-to-head comparison. |
| **Cook**^21^  **2014** | Outcome | Outcome did not evaluate vascular residual stenosis, but instead evaluated individual stent strut expansion and apposition to vascular wall. |
| **Sorrentino**^22^  **2015** | Intervention | Inflation duration for prolonged inflation treatment arm was not greater than 60 seconds. |
|  |  |  |

**Appendix 2**: Reasons for study exclusions.

|  |  |  |  | **Balloon Inflation Protocol** | | | | **Number of Units Analyzed** | | | |
| --- | --- | --- | --- | --- | --- | --- | --- | --- | --- | --- | --- |
|  | **Study Design** | | | **Balloon Inflation Duration (s)** | | **Number of Repeat Inflations** | | **Number of Subjects** | | **Number of Lesions** | |
| **Study** | **Design** | **Setting** | **Arteries** | **Brief** | **Prolonged** | **Brief** | **Prolonged** | **Brief** | **Prolonged** | **Brief** | **Prolonged** |
| **Arie**^5^  **1990** | Before - After Trial | Single Center | Coronary | < 40 | > 60 | 3 - 4 | 3 - 4 | 195 | 244 | 207 | 265 |
| **Ohman**^15^  **1993** | RCT | Multicenter | Coronary | 60 | 900 | 2 - 4 | 1 - 2 | 236 | 242 | 236 | 242 |
| **Eltchaninoff**^13^  **1996** | RCT | Multicenter | Coronary | < 60 | 180 - 300 | 3 - 5 | 3 - 5 | 149 | 140 | 161 | 149 |
| **Zorger**^9^  **2002** | RCT | Single Center | Femoropopliteal | 30 | 180 | 1 | 1 | 37 | 37 | 37 | 37 |
| **Umeda**^6^  **2004** | RCT | Multicenter | Coronary (Small Diameter) | < 60 | > 600 | 1 | 1 | 85 | 90 | 85 | 90 |
| **Cribier**^14^  **1995** | RCT | Unclear | Coronary | < 60 | 120 - 300 | 3 - 5 | 3 - 5 | 22 | 19 | 24 | 20 |

**Appendix 3**: Baseline characteristics of each study, including design, balloon inflation duration protocol, and the number of units analyzed.

|  |  |  | **Immediate Post-Inflation Outcomes** | | | | | | | | |
| --- | --- | --- | --- | --- | --- | --- | --- | --- | --- | --- | --- |
|  | **Mean Total Balloon Inflation Duration (s)** | | **Radiologic** | | | | | | **Clinical** | | |
|  |  |  | **Residual Stenosis** | | | **Major Dissection** | | | **Clinical Outcomes** | | |
| **Study** | **Brief** | **Prolonged** | **Definition** | **Brief** | **Prolonged** | **Definition** | **Brief** | **Prolonged** | **Definition** | **Brief** | **Prolonged** |
| **Arie**  **1990** | 101.2 | 238.8 | >50% | 104 | 84 |  |  |  | Emergency CABG | 16 | 7 |
|  |  |  |  |  |  |  |  |  | Death | 5 | 3 |
| **Ohman**  **1993** | 120 | 900 | >50% | 26 | 12 |  |  |  | Inability to Tolerate Balloon Inflation | 0 | 15 |
|  |  |  | Continuous % Stenosis | 38 +/- 8 | 35 +/-9 |  |  |  | Abrupt vessel closure, MI, CABG, or repeat PCI | 9 | 13 |
| **Eltchaninoff**  **1996** | 198 | 782 | Success (>50% and/or dissection) | 32 | 13 | All Dissection | 48 | 21 | Inability to Tolerate Balloon Inflation | 0 | 19 |
|  |  |  | Continuous % Stenosis | 31 +/- 12 | 28 +/- 12 | Severe Dissection | 15 | 4 |  |  |  |
| **Zorger**  **2002** |  |  | >30% | 12 | 5 | Grade 3 or 4 (NHLBI Criteria) | 16 | 5 | ABI Improvement Immediately Post-Procedure | 0.21 | 0.19 |
| **Umeda**  **2004** | 141 | 973 | >35% | 34 | 16 | Class D or greater (NHLBI) | 17 | 5 | Inability to Tolerate Balloon Inflation | 0 | 5 |
|  |  |  | Continuous % Stenosis | 29.4 +/- 15.2 | 27.4 +/- 10.5 |  |  |  | Periprocedural MI | 3 | 5 |
| **Cribier**  **1995** |  |  | Continuous % Stenosis | 36 +/- 8 | 26 +/- 10 | Identified on Angiography | 4 | 4 |  |  |  |
|  |  |  |  |  |  | Identified on Angioscopy | 16 | 6 |  |  |  |

**Appendix 4**: Immediate results following initial balloon angioplasty. Blank fields represent unreported data.

|  | **Off-Protocol Intraoperative Results** | | | | | |
| --- | --- | --- | --- | --- | --- | --- |
|  | **Adjunctive Procedures** | | | **Residual Stenosis After Adjunctive Procedures** | | |
| **Study** | **Definition** | **Brief** | **Prolonged** | **Definition** | **Brief** | **Prolonged** |
| **Arie**  **1990** |  |  |  | >50% | 43 | 36 |
| **Ohman**  **1993** | Further Inflation | 71 | 43 | >50% | 5 | 5 |
|  |  |  |  | Continuous % Stenosis | 35+/-15.7 | 32+/-17.6 |
| **Eltchaninoff**  **1996** | Further Inflation and/or Stent | 32 | 12 | Failure (>50% and/or dissection) | 21 | 13 |
|  |  |  |  | Continuous % Stenosis | 31+/-12 | 28+/-12 |
| **Zorger**  **2002** | Stent | 4 | 1 |  |  |  |
|  | Further Inflation | 16 | 8 |  |  |  |
| **Umeda**  **2004** | Stent | 20 | 5 | >35% | 4 | 2 |
| **Cribier**  **1995** |  |  |  |  |  |  |

**Appendix 5**: Off-protocol intraoperative results of any adjunctive procedures following initial angioplasty. Blank fields represent unreported data.

|  | **Long-Term Results** | | | | | | | | | |
| --- | --- | --- | --- | --- | --- | --- | --- | --- | --- | --- |
|  | **Follow-Up Characteristics** | | | | **Radiologic** | | | **Clinical** | | |
|  |  |  | **Number Re-Imaged** | | **Residual Stenosis** | | | **Clinical Outcomes** | | |
| **Study** | **Follow-Up Timeline** | **Type of Imaging** | **Brief** | **Prolonged** | **Definition** | **Brief** | **Prolonged** | **Definition** | **Brief** | **Prolonged** |
| **Arie**  **1990** |  |  |  |  |  |  |  |  |  |  |
| **Ohman**  **1993** | 6 - 12 Months | Angiogram | 145 | 174 | >50% | 64 | 77 |  |  |  |
|  |  |  |  |  | Continuous % Stenosis | 35 +/- 18 | 32 +/- 18 |  |  |  |
| **Eltchaninoff**  **1996** | 4 - 6 Months | Angiogram | 123 | 116 | >50% | 52 | 54 | >2 CCF Class | 11 | 14 |
|  |  |  |  |  | Continuous % Stenosis | 48 +/- 22 | 47 +/- 22 |  |  |  |
| **Zorger**  **2002** |  |  |  |  |  |  |  |  |  |  |
| **Umeda**  **2004** | 6  Months | Angiogram | 85 | 83 | >50% | 43 | 26 | MACE | 35 | 19 |
|  |  |  |  |  | Continuous % Stenosis | 51.8 +/- 21.7 | 45.4 +/- 19.5 |  |  |  |
| **Cribier**  **1995** |  |  |  |  |  |  |  |  |  |  |

**Appendix 6**: Long-term results following balloon angioplasty. Blank fields represent unreported data.
